# Machine learning to identify suitable boundaries for band-pass spectral analysis of dynamic [$^{11}$C]Ro15-4513 PET scan and voxel-wise parametric map generation

**DOI:** 10.1186/s13550-025-01251-5

**Published:** 2025-07-11

**Authors:** Zeyu Chang, Colm J. McGinnity, Rainer Hinz, Manlin Wang, Joel Dunn, Ruoyang Liu, Mubaraq Yakubu, Paul Marsden, Alexander Hammers

**Affiliations:** 1 School of Biomedical Engineering and Imaging Sciences, King’s College London and Guy’s and St Thomas’ PET Centre, King’s College London, Westminster Bridge Rd, London, SE1 7EH UK; 2 Wolfson Molecular Imaging Centre, University of Manchester, 27 Palatine Rd, Manchester, M20 3LJ UK; 3 School of Biomedical Engineering and Imaging, King’s College London, Lambeth Palace Rd, London, SE1 7EU UK; 4 Business and Tourism Institute, Hangzhou Vocational and Technical College, Xueyuan St, Hangzhou, 310018 China

**Keywords:** Band-pass spectral analysis, [$^{11}$C]Ro15-4513, $\hbox {GABA}_\text {A}$, $\alpha$5, PET, Parametric map

## Abstract

**Background:**

Spectral analysis is a model-free PET quantification technique that treats the time-space signal as an impulse response to a bolus injection. Band-pass spectral analysis, considering specific frequency ranges, enables calculation of separate parametric maps of receptor subtype tracer binding for suitable radiopharmaceuticals such as [$^{11}$C]Ro15-4513 binding to GABA_A_ $\alpha$1/5 subunits. Frequency ranges are based on inspection of spectra, prior knowledge of receptor distribution, and blocking studies. The process currently requires the manual selection of frequency ranges based on the data. To enhance the efficiency of band-pass spectral analysis and extend its application to a broader range of tracers, we propose employing machine learning to automate the selection of spectral boundaries. Based on these boundaries, voxel-wise parametric maps can be generated. The machine learning models utilized in this study include 1D Convolutional Neural Network, Neural Network, Support Vector Machine, Logistic Regression, K-nearest neighbors, and Fine Tree.

**Results:**

The best machine learning model, Fine Tree, agreed with the manual frequency boundary in 96.92% of 3185 ROIs. The absolute mean error was 3.80% for slow component volume-of-distribution ($\hbox {V}_{slow}$, largely representing $\alpha$5) and 4.74% for fast component volume-of-distribution($\hbox {V}_{fast}$, largely representing $\alpha$5), while the relative error was 2.83% ± 43.47% for $\hbox {V}_{slow}$ and $-$2.01% ± 78.04% for $\hbox {V}_{fast}$. The median test-retest intraclass correlation coefficient across six representative regions was 0.770 for $\hbox {V}_{slow}$, 0.670 for $\hbox {V}_{fast}$, and 0.502 for total component volume-of-distribution($\hbox {V}_d$). Parametric maps applying different boundaries for different ROIs were generated.

**Conclusion:**

The machine learning model developed provided accurate boundary predictions in 96.92% of regions, with minimal average bias. However, when errors occur, they can be large, owing to the sparsity of peaks. The model enables setting boundaries automatically for the vast majority of regions, followed by manual checking of the outliers. It opens the possibility of accelerating analyses e.g. of GABA_A_ $\alpha$1/2/3/5 subunit binding using [^11^C]flumazenil and of extending band-pass spectral analysis to other receptor systems.

## Introduction

      Positron Emission Tomography (PET) is a three-dimensional computed tomo-graphy imaging technique with picomolar sensitivity. The principle involves injecting appropriate molecules labelled with positron-emitting radionuclides into the patient; for the brain, neurotransmitter receptors are frequently targeted. As the radionuclides undergo positron decay, they emit pairs of annihilation photons. By detecting these photons, a concentration map of the tracers within the target organ is generated. This allows for the inference of the distribution of the coupled molecules. Dynamic PET scanning is a powerful technique for imaging and quantifying neurotransmitter receptor concentrations in the brain. Dynamic PET imaging involves recording multiple time frames over a period following the injection of the tracer into the patient. During this process, regular blood sampling is performed to obtain the metabolite-corrected parent plasma arterial input function, which is essential for quantifying the dynamic PET data. Due to its reliance on arterial input data collection, fully quantified dynamic PET is less commonly used in clinical applications and is more prevalent in research contexts. These include drug development, the development of new tracers, and neuroscience research, such as studies on the gamma-aminobutyric acid (GABA) receptor system in the human brain.

Machine learning [1, 2] is a technology that leverages algorithms and statistical models to analyse patterns in data and make predictions, enabling decision-making with minimal human intervention. Previously, machine learning has been employed in spectral analysis, playing a significant role in fields such as physics [3], analytical chemistry [4], medicine [5], and biology [6]. However, its application in the quantification of dynamic PET data remains unexplored.

In the quantification of dynamic PET, parameter derivation typically necessitates the a priori specification of a model structure, such as compartmental modeling [7]. Compartmental modeling involves a series of physiological assumptions about the neurotransmitter receptor systems in the brain [8], such as the free exchange of radioactive ligands between compartments and arterial plasma, and that equilibrium is rapidly reached by nonspecifically bound radioactivity in the compartments and in free tissue. However, not all receptor systems conform to these assumptions, and selecting an appropriate compartmental model for a novel receptor system can be challenging.

Spectral Analysis in PET quantification [9] is a model-free quantification technique that considers the temporospatial signal as an impulse response to the bolus injection (blood activity curve). The tissue response reflected in the spectrum typically consists of several kinetic components, with very fast components representing e.g. the rapid inflow and outflow of tracer; very slow components (close to the limit imposed by the isotope half-life) representing irreversible binding; and intermediate components representing target binding (tissue retention and release of tracer). Spectral analysis has proven the most reliable and reproducible quantification method in test-retest studies of various radiotracers [10–13] and has shown superior detection of clinically relevant abnormalities [14].

The application of spectral analysis to dynamic PET data involves deconvolving the time-activity curve (TAC) of the region of interest (ROI) by the arterial input function. Subsequently, a set of exponential basis functions is used to describe the impulse response function (IRF), and the model is fitted using the non-negative least squares (NNLS), as shown in formula 1 [15, 16].

1 $$\begin{aligned} C_{tiss}(t_j)= \sum _{i=1}^{k} \alpha _i\cdot C_m(t)\otimes exp(-\beta _i\cdot t) \end{aligned}$$

Here, $\hbox {C}_{tiss}$(t) represents the time-activity function of the region of interest, $\hbox {C}_m$(t) represents the plasma input function, k represents the maximum number of exponential terms, typically set to 100. $\alpha$ represents the magnitude of the spectral exponential terms, and is non-negative. $\beta$ represents the frequency of the spectral exponential terms obtained, covering a range from the slowest radioactive decay (related to the half-life of the radionuclide) to instantaneous phenomena.

After solving using NNLS, among the k exponential terms, most values are 0, with only a few terms having positive values. While it was originally thought that the various components could not be interpreted in biologically meaningful ways, research over the last 15 years further described below has shown that some may represent receptor subtypes with different kinetic properties. Myers and colleagues discovered that when performing spectral analysis on [$^{11}$C]Ro15-4513, which primarily binds to the $\hbox {GABA}_\text {A}$ receptor subtypes $\alpha$1 and $\alpha$5, two distinct peak groups appeared in the results. When zolpidem was used to selectively block binding to the $\alpha$1 subtype, the spectral analysis resulted in only one peak group remaining [17]. Thus, spectral analysis serves as a crucial tool for studying the distribution of different receptor subtypes in the brain from a single injection of a radiopharmaceutical with more than one, kinetically distinguishable, binding site.

Since spectral analysis is able to distinguish receptor subtypes based on their kinetic characteristics, Myers and colleagues proposed the concept of band-pass spectral analysis for use with [$^{11}$C]Ro15-4513 [17]. Specifically, [$^{11}$C]Ro15-4513 binds to two different receptor subtypes, $\alpha$1 and $\alpha$5, that exhibit distinct kinetic characteristics (with $\alpha$5 having slower kinetics than $\alpha$1), allowing the use of a band-pass filter to isolate kinetic components within a specific spectral range. The components within the passband share similar kinetic characteristics and can be considered as representing the same receptor subtype. By combining a priori physiological knowledge about the distribution of different receptor subtypes with spectral analysis data from different brain regions, and the results from blocking studies with pharmacological agents selectively blocking only one of the binding sites [17], it is possible to set different band-pass filters for various receptor subtypes. This approach enables the determination of the distribution of specific receptor subtypes within the brain.

Previous studies first tested band-pass spectral analysis in the GABA receptor systems. GABA is the primary and most widely distributed inhibitory neurotransmitter in the mammalian brain [18]. GABA receptors primarily consist of three types: $\hbox {GABA}_\text {A}$, $\hbox {GABA}_\text {B}$, and $\hbox {GABA}_\text {C}$ receptors. Among them, $\hbox {GABA}_\text {A}$ and $\hbox {GABA}_\text {C}$ receptors belong to the ligand-gated chloride ion channel superfamily, mediating fast inhibitory responses, while $\hbox {GABA}_\text {B}$ receptors are members of the large GTP-binding protein-coupled receptor family, controlling $\hbox {Ca}^\text {2+}$ and $\hbox {K}^\text {+}$ channels and mediating long-term inhibitory responses [19].

The $\hbox {GABA}_\text {A}$ receptor comprises at least 19 subunits: $\alpha$1–6, $\beta$1–3, $\gamma$1–3, $\rho$1–3, as well as $\sigma$,$\epsilon$, $\pi$, and $\theta$, leading to a high degree of heterogeneity in $\hbox {GABA}_\text {A}$ receptor subtypes. However, most receptor subtypes contain $\alpha$, $\beta$, and $\gamma$ subunits. Among these receptor subtypes, the three most common subtypes in the central nervous system are $\alpha _1\beta _2\gamma _2$, $\alpha _3\beta _3\gamma _2$, and $\alpha _2\beta _3\gamma _2$ [20]. The multitude of $\hbox {GABA}_\text {A}$ receptor subtypes provides a basis for the diverse modulation of GABA signaling and drug modulation (for example, with benzodiazepines, barbiturates, and neurosteroids) in the central nervous system.

Currently, there is no precise description of the distribution of all $\hbox {GABA}_\text {A}$ receptor subunits in the brain. However, based on immunocytochemical data from rodents and mRNA data of receptor subunits, it is generally believed that in the adult mammalian brain, the $\alpha$1, $\beta$2, $\beta$3, and $\gamma$2 subunits are widely distributed in the same pattern, while the remaining receptor subunits are only found in certain brain regions [21]. $\alpha$5 is distributed in memory-related brain regions such as the hippocampus, associated with cognitive, learning, and memory functions, and generally in limbic areas. With the application of band-pass spectral analysis to suitable radiopharmaceuticals beyond Ro15-4513, it may be possible to separate these receptor subtypes based on their dynamic characteristics, which would facilitate in-depth research on the distribution and functional characteristics of individual receptor subtypes [21].

Ro15-4513 ($C_{15}H_{14}N_6O_3$) is a partial inverse agonist of the $\hbox {GABA}_\text {A}$ benzodiazepine receptor, primarily binding to receptor subtypes containing the $\alpha$5 subunit (with inhibition constant [Ki] of approximately 0.7 nmol/L), exhibiting affinity 105 times higher compared to receptor subtypes containing the $\alpha$1, $\alpha$2and $\alpha$3 subtype (with Ki values approximately 7–10 nmol/L) [22, 23]. However, due to the higher abundance of the $\alpha$1 subtype compared to the $\alpha$5 subtype in the central nervous system (CNS), the scanning signal of [$^{11}$C]Ro15-4513 is typically considered to have components from both the $\alpha$1 and $\alpha$5 subtypes [24].

Previous studies have elucidated that spectral analysis of PET scans using [$^{11}$C]Ro15-4513 as a tracer can yield two groups of peaks within the spectrum. Based on prior knowledge of receptor distribution and blocking studies [17], the peak representing slower components is believed to correspond to binding to the $\alpha$5 subtype, while the peak representing faster components is associated with binding to the $\alpha$1 subtype [17]. To distinguish between signals from the two subtypes, Myers et al. [25] proposed band-pass spectral analysis to differentiate binding with distinct kinetic characteristics with a band-pass spectral range for the slow component of 0.0006371–0.001 ${\textrm{s}}^{-1}$ out of a total range of 0.0006371–1 ${\textrm{s}}^{-1}$. Subsequently, McGinnity et al. [12]set a spectral boundary at 0.00137 ${\textrm{s}}^{-1}$ within the spectral range to differentiate between the two groups of kinetic components largely representing $\alpha$1 and $\alpha$5, thereby generating separate parametric maps for $\alpha$1 and $\alpha$5 binding with the spectral range of 0.0006371–0.00137 ${\textrm{s}}^{-1}$ ($1\textrm{st}$ bin to $15{\textrm{th}}$ bin) representing $\alpha$5 binding and 0.00137–0.1 ${\textrm{s}}^{-1}$ ($15{\textrm{th}}$ bin to $100{\textrm{th}}$ bin) representing $\alpha$1 binding. Finally, parametric maps based on fixed spectral boundaries were generated.

The entire spectral range was evenly divided into 100 bins to facilitate the description of peak regions and to distinguish between fast and slow components more effectively.

We have observed that the positions and heights of bins from spectral analysis vary across subjects and regions in both literature and our data, therefore, either of the above choices of band-pass frequency ranges has limitations, as using the same band-pass range for different subjects and brain regions can introduce errors. To more accurately separate the two receptor subtypes by adapting the boundaries to variations in ROI and subject, manual selection of the frequency range would be required, which is highly labour-intensive. To address these issues, this study introduces the concept of machine learning to select appropriate spectral boundaries for different brain regions and participants individually, thereby aiming to improve the performance and applicability of band-pass spectral analysis.

The main contributions of this study are as follows:

1. Introduction of machine learning into PET spectral analysis, automating the selection of band-pass spectral boundaries across regions and subjects.
2. Validation of the accuracy of boundary selection with machine learning models using test-retest data.
3. Utilization of the model-generated band-pass spectral boundaries to produce voxel-wise parametric distribution maps for single receptor subtypes.

## Material and methods

### Subjects

      In this study, we quantified 40 dynamic [$^{11}$C]Ro15-4513 PET scan datasets from 35 volunteers [12]: 12 individuals with epilepsy recruited from outpatient epilepsy clinics at the National Hospital for Neurology and Neurosurgery (Queen Square, London) and the Chalfont Centre for Epilepsy (Chalfont St Peter) (median age ± interquartile range 40 ± 7.5 years, 7 males, 9 right-handed, seizure-free interval 6 ± 25 days, all on medication which can suppress seizures); 17 healthy male controls from a previous study [26]; and 6 additional healthy male controls, of whom 5 completed retest scans on the same scanner [11].

### Dynamic PET and MRI acquisition

      The dynamic PET scans were acquired in 3D mode on a Siemens/CTI ECAT EXACT HR+ 962 scanner. Each participant underwent a 10-minute transmission scan for attenuation correction, followed by a 90-minute dynamic scan comprising 24 frames (1 $\times$ 30, 4 $\times$ 15, 4 $\times$ 60, 2 $\times$ 150, 10 $\times$ 300, 3 $\times$ 600 s). At 30 s after the start of the scan, [$^{11}$C]Ro15-4513 was administered as a bolus injection over 30 s via intravenous infusion. Throughout the scan, continuous monitoring for evidence of seizures in temporal lobe epilepsy (TLE) patients was conducted (none detected). Frames were reconstructed using Fourier rebinning [27] and 2D filtered backprojection (ramp filter, kernel 2.0 mm full-width at half-maximum) into 63 transaxial images.

All participants underwent 3D T1-weighted MRI scans, with approximately millimeter-scale isotropic voxels. These scans were utilized for the co-registration of dynamic PET images and for defining brain regions to collect TACs.

### Input function

      Before the experiment, a radial artery cannula was inserted into the non-dominant wrist’s radial artery of each participant. Baseline discrete blood samples were collected prior to the scan initiation. During the first 15 min of the experiment, continuous monitoring of blood radioactivity was conducted using a bismuth germanate detection system [28]. At 10 intermittent time points following the commencement of the scan, 10 ml discrete blood samples were collected for quantifying plasma and whole-blood radioactivity. The parent fraction of the radiotracer was quantified via high-performance liquid chromatography. Plasma-to-blood ratio and metabolite models were fitted for across the continous blood measurements from 0 to 15 min and the discrete measurements up to 90 min. Continuous parent plasma input functions were derived using the in-house software Clickfit run in MATLAB R2014a (The MathWorks, Natick, MA, USA), as previously described [10, 11, 13, 29].

### Pre-processing and spectral analysis

      Spectral Analysis was performed within the the in-house software MICK and MICKPM (Author: Rainer Hinz, rainer.hinz@manchester.ac.uk). Before applying spectral analysis, the PET frames were first de-noised using wavelets [30]. The MRIs were registered to the summed PET images following these steps:

Firstly, a summed image of the dynamic PET was created using MICKPM based on MATLAB as a reference image for registration, against which T1-weighted MRI images were co-registered.

Subsequently, anatomical segmentation of the T1-weighted images was performed using MAPER (multi-atlas propagation with enhanced registration [31]) to delineate ROIs in the brain. High-dimensional image registration was used to propagate 30 MRI datasets (each associated with manually delineated labels of 95 regions https://brain-development.org/brain-atlases/) [32–36] to the target brain. Label fusion was utilized to obtain images consisting of 95 labeled ROIs in the target space [37].

Finally, the anatomical images were used as masks to extract STATS files for the 95 ROIs using the fslstats command(https://fsl.fmrib.ox.ac.uk/fsl/fslwiki/FSL). These STATS information files comprised calculations of TAC for each voxel within the ROI, where the mean TAC of each ROI was utilized for subsequent spectral analysis. Concurrently, continuous parent plasma input functions without decay correction were used to generate impulse response functions for the 91 regions. The corpus callosum (white matter), lateral ventricle and third ventricle (cerebrospinal fluid) were excluded due to weak signals and significant noise interference.

We applied weights [11] to the TACs of each ROI and voxel as formula 2:

2 $$\begin{aligned} W_{i}=L_{i}/T_{i} \, (i=1,2,3,...,24;non-decay-corrected) \end{aligned}$$

where:

$\hbox {W}_i$ is the weight of the i-th frame.

$\hbox {L}_i$ is the length of the i-th frame in seconds.

$\hbox {T}_i$ is the real coincidences count rate of the i-th frame in counts per second.

The weights are normalized such that the sum of weights equals 24.

We excluded regions primarily composed of white matter and cerebrospinal fluid. Therefore, each participant had spectral analysis results for 91 ROIs, resulting in a total of 3185 ROI spectral analysis results for training and validation of the machine learning model from the 35 patient and first (test) control datasets. Each spectral analysis result comprised 100 feature channels corresponding to the 100 exponential terms and their respective amplitudes, with most of the data in the 100 features being 0, as described above. The amplitudes were computed to obtain volume of distribution ($\hbox {V}_d$) values using formula 3 [38].

3 $$\begin{aligned} V(t)=\sum _{i=1}^{k}\alpha _i/(\beta _i-\lambda ) \end{aligned}$$

Here, $\lambda$ represents the radioactive half-life of the radionuclide of the element, k represents the maximum number of exponential terms: 100, $\alpha$ and $\beta$ represent the magnitude and frequency of the spectral exponential terms obtained which are non-negative. For [$^{11}$C]Ro15-4513 in formula 1, $\beta$ range spans from 0.00063–0.1 ${\textrm{s}}^{-1}$. Variable t indicates the function’s value at particular times, or the function’s value derived from integral data gathered during specific time frames.

Furthermore, the identifier numbers of ROIs were used as an additional feature for model construction. Consequently, the data used for model training and validation consisted of a 2D array with dimensions 3185*101.

### Ground truth label setting

      Although there is no gold standard for boundary setting, previous studies utilizing zolpidem blocking [17] have provided valuable insights into distinguishing between $\alpha$1 and $\alpha$5 peaks. Zolpidem selectively blocks $\alpha$1 without affecting $\alpha$5, which is reflected in the frequency domain as a reduction in the peak representing $\alpha$1, while the peak corresponding to $\alpha$5 remains largely unchanged.

**Fig. 1:**
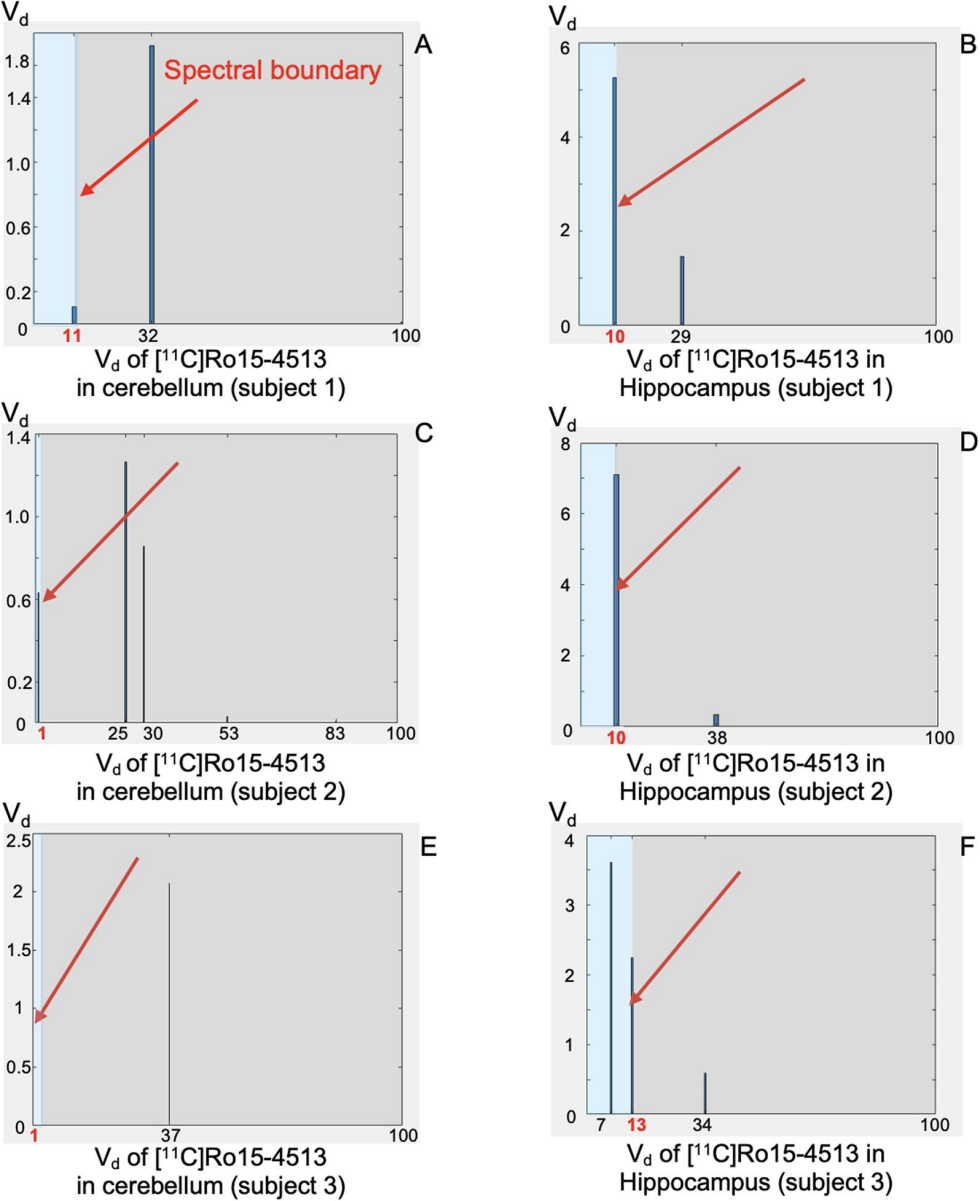
Volume of distribution of [$^{11}$C]Ro15-4513 of cerebellum ($\alpha$5 rich) in subject 1(**A**), subject 2(**C**) and subject 3(**E**), volume of distribution of [$^{11}$C]Ro15-4513 of hippocampus ($\alpha$5 rich) in subject 1(**B**), subject 2(**D**) and subject 3(**F**), where $1\textrm{st}$bin-$100{\textrm{th}}$bin corresponding to $\beta$ 0.000631 to 0.1 $\hbox {seconds}^{-1}$. The arrows in the figure indicate the spectral boundaries. X axes are equalised for all panels, but Y axes have different ranges for better visualisation of peak height differences

In the spectral analysis results of [$^{11}$C]Ro15-4513, multiple spectral components are typically observed within the defined frequency range, representing different kinetic components. Both the peak heights and positions of these spectral components vary across ROIs and subjects, as shown in Fig. 1. The cerebellum, being an $\alpha$5-poor ROI (Fig. 1a, c, e), and the hippocampus, being an $\alpha$5-rich ROI (Fig. 1b, d, f), exhibit distinct patterns. Generally, in the cerebellum, the spectral analysis results show either the absence of the $\alpha$5 peak (e.g., Fig. 1e) or a very weak signal (e.g., Fig. 1a, c). In contrast, the hippocampus exhibits strong $\alpha$5 signals.

Moreover, a single receptor subtype is not necessarily represented by a single peak within the spectral range; multiple peaks may appear (e.g., Fig. 1f). Consequently, we selected the fastest slow component within the spectral range as the boundary. For spectra without slow components, we chose $1\textrm{st}$ bin as the boundary. Based on our observations, the fastest slow component across 3185 cases was consistently located within the first 19 bins. Therefore, no spectral boundaries beyond $19{\textrm{th}}$ bin are present in our results.

We manually set the spectral boundaries across ROIs and subjects based on the following evidence: 1. Prior physiological knowledge of ROIs. 2. Observational experience with nearly 4000 spectral analysis results. 3. Previous boundary settings from previous spectral analysis studies.

The peak height and position of the spectral peaks vary for each ROI; however, the distribution patterns are similar. For instance, in the hippocampus, there is consistently a particularly high slow peak representing $\alpha$5, along with a relatively lower fast peak representing $\alpha$1. Prior knowledge, along with the results from 40 scans, helped us determine the spectral peak distribution trend for each ROI, which can be used to validate whether the spectral boundary settings are correct. To minimize potential errors, we re-annotated all spectral analysis results five times, when the boundary settings differed, a re-evaluation and adjustment were considered accordingly.

**Fig. 2:**
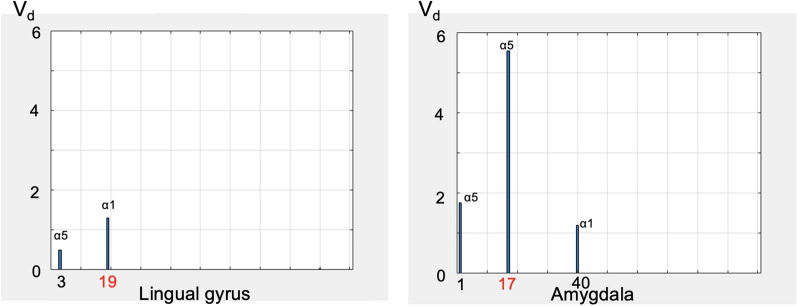
Volume of distribution of [$^{11}$C]Ro15-4513 of lingual gyrus ($\alpha$5 poor) and amygdala ($\alpha$5 rich) in single subject. Both X axes and Y axes are equalised for both panels

In most cases, the slow component representing $\alpha$1 appears to the right of bin 19, however, it may also occasionally appear to the left of bin 19. As shown in the example in Fig. 2, both the lingual gyrus and the amygdala exhibit a spectral peak near bin 19. The peak in the lingual gyrus occurs at bin 19, which could lead to potential misclassification between $\alpha$1 and $\alpha$5. However, the lingual gyrus is an $\alpha$5-poor region, and the peak in this area is relatively low. Therefore, despite its position within the possible spectral range of $\alpha$5, we classified it as $\alpha$1. In contrast, the peak in the amygdala is located at bin 17 and has a higher peak amplitude, making it more likely to represent $\alpha$5. Additionally, since the amygdala is an $\alpha$5-rich region, we classified this peak as $\alpha$5.

### Models for determining boundaries

**Fig. 3:**
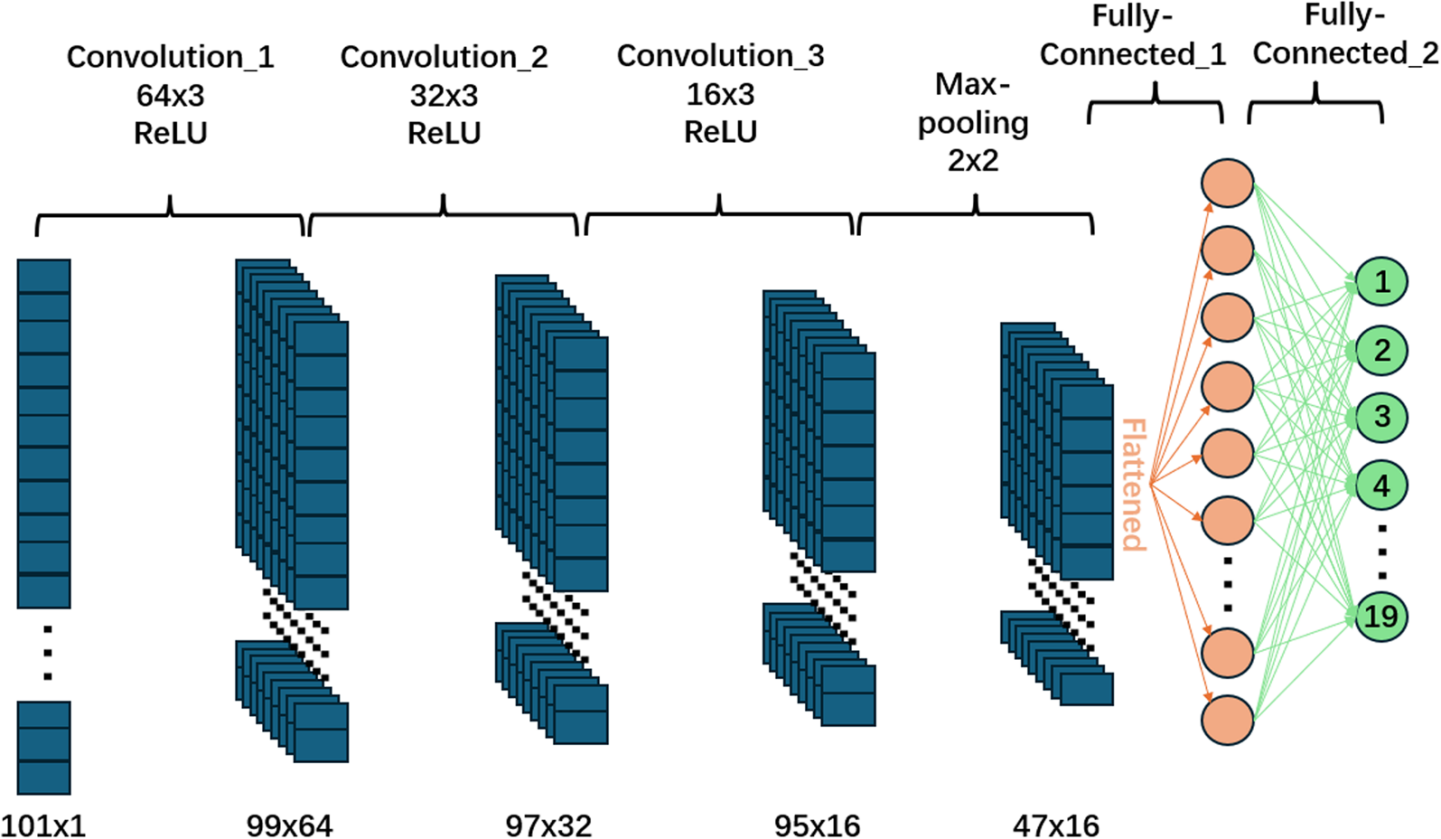
Structure of the CNN used in this study. ReLU, rectified linear unit

      We implemented and evaluated a large number of machine learning models for the automated determination of spectral boundaries in our Spectral Anlalysis data. These were: Neural Network, Efficient Linear Support Vector Machine (SVM), Efficient Logistic Regression, K-Nearest Neighbors (KNN), and Tree-based models. The Neural Network category was further divided into Trilayered, Bilayered, Wide, Medium, and Narrow architectures. The KNN models included Weighted, Cubic, Cosine, Coarse, Medium, and Fine variants. Similarly, the Tree-based models were classified into Coarse, Medium, and Fine. These models were sourced from the Classification Learner application in MATLAB (https://uk.mathworks.com/help/stats/classificationlearner-app.html). In addition to simple classifiers and neural networks from MATLAB, we also trained a 1D data classification model based on a Convolutional Neural Network (CNN), which is shown in Fig. 3.

By comparing the validation accuracy and runtime of different models in selecting spectral boundaries, we determined the most suitable model for automated spectral boundary selection. Due to the relatively simple data structure with only 101 feature channels, most of which had values of 0, we hypothesized that using simpler classifiers such as tree models would achieve high classification accuracy while maintaining a short runtime.

To enhance the robustness of the model accuracy, 5-fold cross-validation was adopted. In each training and validation iteration, 80% of the 3185 spectral analysis results were used for training, while the remaining 20% were used for validation. The selection of training and validation sets was random for each time, and it would be reselected in each training iteration with replacement.

Finally, data from the retest cohort served as the test set for the model. We manually labelled the test data in the same way as the training and validation data to enable calculation of the test accuracy, to evaluate the robustness and reliability of the model’s performance in real-world applications.

Additionally, to eliminate potential bias from using only data from the retest cohort as the test set, given that the test data from these subjects were included in the model’s training set, we also tested the machine learning model’s accuracy by randomly selecting 5 subjects as the test set, while using the remaining subjects for training and validation.

To evaluate the impact of our $101\textrm{st}$ feature, namely the ROI information, on the model’s performance, we also tested the classification accuracy using only the first 100 features.

The purpose of setting spectral boundaries is to separate fast components from slow components, allowing for the independent calculation of their distribution volumes. Therefore, we also evaluated the errors in distribution volume calculations caused by model-generated boundaries compared to manually annotated boundaries.

### Parametric map generation

      Unlike the previous quantification of $\hbox {V}_d$ from region-averaged TACs, here, quantification was conducted voxel-by-voxel. Based on the machine learning model, spectral boundaries were set separately for each of the 91 ROIs in each image. We then performed band-pass spectral analysis with the spectral boundary for voxels in each ROI and computed parametric images for $\alpha$1 (fast component volume-of-distribution: $\hbox {V}_{fast}$) and $\alpha$5 (slow component volume-of-distribution: $\hbox {V}_{slow}$) separately. After combination of all regions, we thus obtained two parametric maps"($\hbox {V}_{fast}$ and $\hbox {V}_{slow}$)"for each subject.

Using the fact that five subjects were scanned twice (test-retest), we calculated the intraclass correlation coefficient (ICC; [39]) to verify the test-retest reliability of the parametric maps supported by the machine learning model using formula 4.

4 $$\begin{aligned} ICC=\frac{MSBS-MSWS}{MSBS+(dfWS\times MSWS)} \end{aligned}$$

      Here, MS represents the mean sum of squares; BS denotes between-subject; WS refers to within-subject; and df indicates the degrees of freedom.

The closer the ICC is to 1, the smaller the test-retest variability compared with the intersubject variability and the more reliable the retest. In this study, the"one-way random"model in SPSS [40] was used to calculate the"single measure"ICC. We identified regions for ICC assessment based on areas expected to have high concentrations of the $\alpha$5 subtype: high-concentration limbic regions: anterior cingulate gyrus (ACGs) and hippocampus; intermediate concentration regions: fusiform (occipitotemporal) gyrus, inferior frontal gyrus, and insula; and a low concentration region, the occipital lobes. Among these, the insula is represented by five regions in the 95-region map, and the occipital lobes are represented by three regions. The volume of distribution for these regions was obtained by averaging across these regions. All regions considered included both the left and right hemisphere counterparts.

We also compared the ICCs of fixed boundaries (with the values used in McGinnity CJ et al. 2021 [12]), the manual boundaries used as ground truth in this study, and the AI-generated boundaries established in this study.

## Results

### Model evaluation

**Fig. 4:**
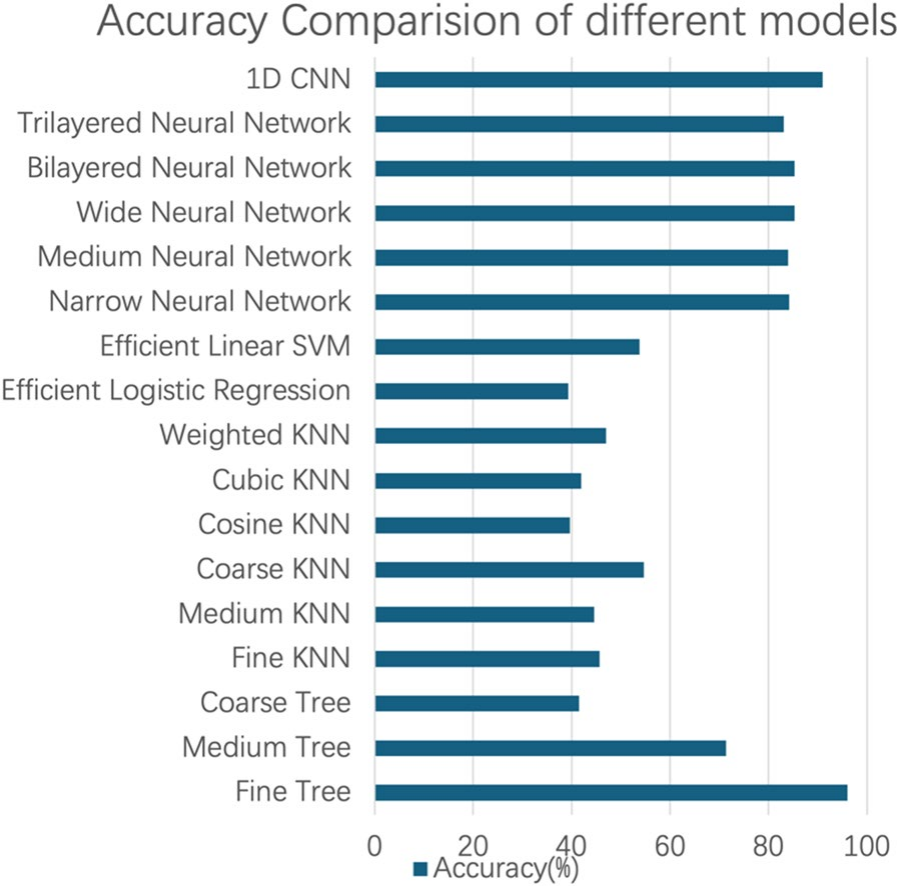
Comparison of the fivefold average accuracy of different models (SVM:support vector machines, KNN:k-nearest neighbor)

      In this section, we present a performance comparison of different machine learning models in the task of spectral boundary selection. A list of the models and their accuracy is shown in Fig. 4.

The Fine Tree model achieved the highest accuracy at 96.92%, followed by the deep learning network 1D CNN model with an accuracy of 91.00%. Other neural network models and the Medium Tree model also demonstrated good accuracy, while the remaining models were not effective in selecting the boundaries. When randomly selecting 5 subjects as the test set, while the remaining subjects were used for training and validation, the model achieved a similar accuracy of 97.36%. When we trained the machine learning model using 100 features instead of 101, the accuracy was 95.82%, slightly lower than the 96.92% accuracy achieved with 101 features.

**Fig. 5:**
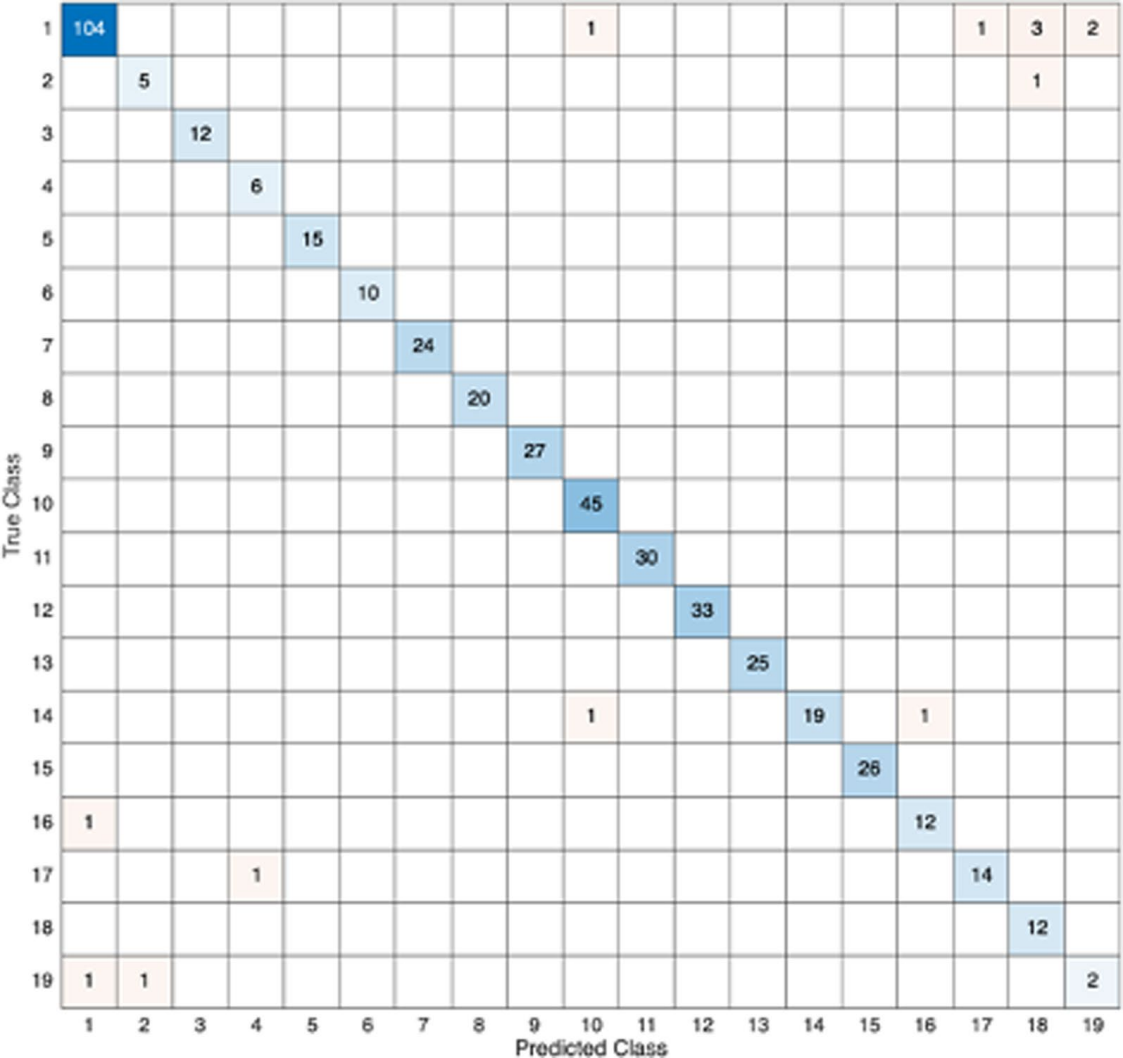
Confusion matrix for Fine Tree boundary selection. The bins shown in the figure are spectral boundaries and represent the fastest slow peaks. Only bins 1–19 (out of 100) are shown as 19 was the fastest slow peak in the dataset

To further analyse the reasons for remaining misclassification in the Fine Tree model, a confusion matrix was calculated in Fig. 5. The classifier ultimately regressed to 19 categories because the model assumes that slow components only exist within $1\textrm{st}$ bin to $19{\textrm{th}}$ bin, while any components beyond this range are considered as fast components.

We also examined the correlations within-subject between-regions and within-region between-subjects. The Pearson correlation coefficient of within-subject between-regions was 0.015 ($p<$ 0.750), the Pearson correlation coefficient of within-regions between-subjects was 0.158 ($p<$ 0.001).

### Region-based volumes-of-distribution

      We calculated the absolute mean error and relative error for the volume-of-distribution based on model-generated boundaries. The mean absolute error was 3.8% for $\hbox {V}_{slow}$ and 4.7% for $\hbox {V}_{fast}$, while the relative error was 2.8% ± 43.5% for $\hbox {V}_{slow}$ and $-$2.0% ± 78.0% for $\hbox {V}_{fast}$.

### Parametric maps

**Fig. 6:**
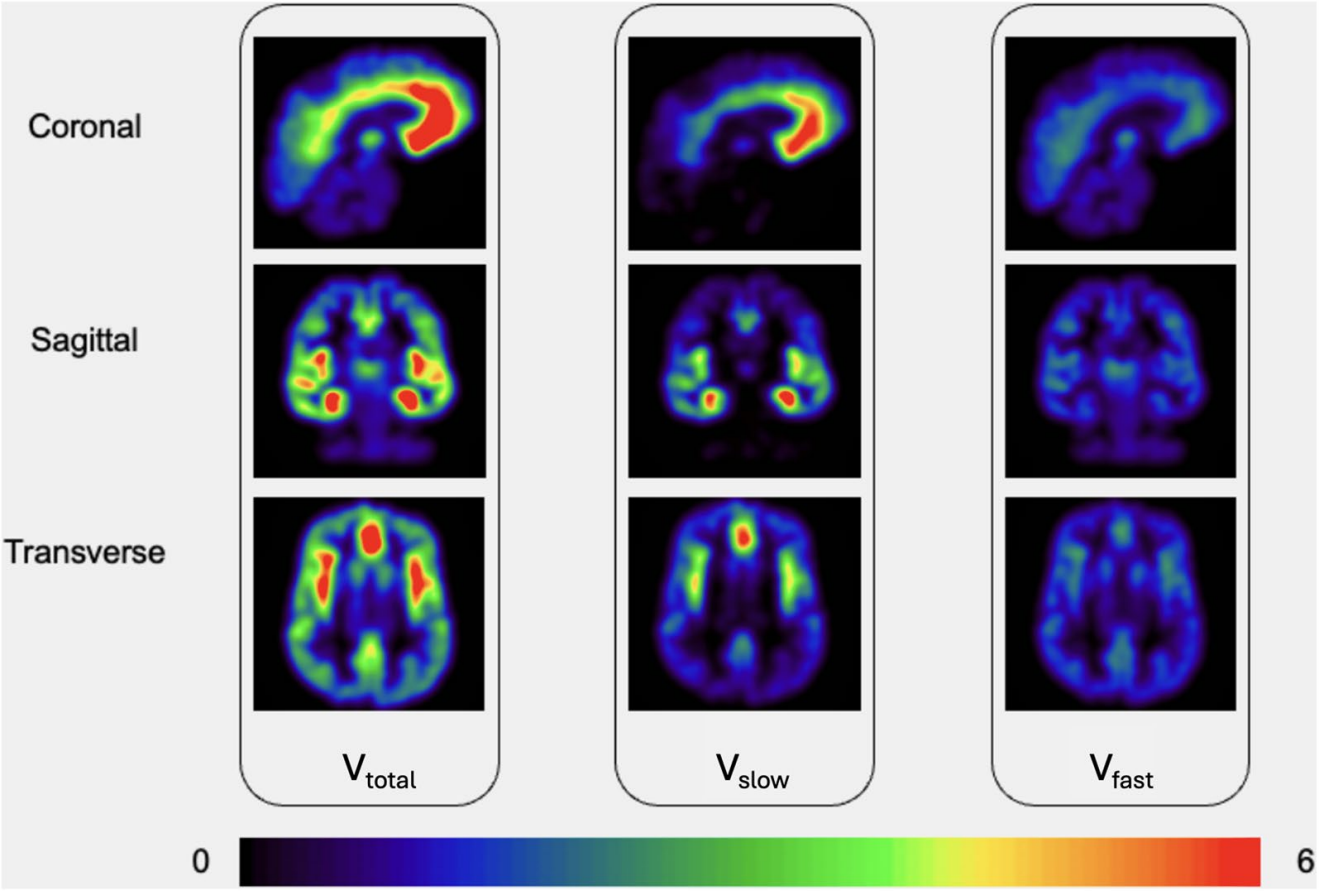
Parametric Maps of $\hbox {V}_{slow}$, $\hbox {V}_{fast}$, $\hbox {V}_{total}$ generated by automatically set boundaries. $\hbox {V}_{total}$ represents the total distribution volume, $\hbox {V}_{fast}$ represents the fast component fraction (corresponding broadly to the distribution volume of $\alpha$1) and $\hbox {V}_{slow}$ represents the slow component fraction, corresponding broadly to the distribution volume of $\alpha$5. $\hbox {V}_{slow}$ shows a strong signal in limbic areas such as the hippocampus and amygdala. C exhibits a signal that is more dispersed throughout the entire brain. $\hbox {V}_{total}$ is the sum of $\hbox {V}_{slow}$ and $\hbox {V}_{fast}$

      The parametric maps of the two different kinetic components of [$^{11}$C]Ro15-4513 obtained by applying the spectral boundaries automatically selected by the above-mentioned machine learning model to different regions of the brain for band-pass spectral analysis of one of the healthy controls from Hammers Group [11] are shown in Fig. 6.

It can be observed that $\hbox {V}_{slow}$, which corresponds to $\alpha$5, shows a strong signal in limbic areas such as the hippocampus and amygdala. On the other hand, $\hbox {V}_{fast}$, representing $\alpha$1, exhibits a signal that is more dispersed throughout the entire brain. Similarly, total component volume-of-distribution ($\hbox {V}_{total}$), like $\hbox {V}_{slow}$, shows a strong signal in the limbic areas, as [$^{11}$C]Ro15-4513 primarily binds to $\alpha$5, which predominantly reflects the distribution of $\alpha$5.

The ICCs of fixed boundaries, manual boundaries, and AI-generated boundaries are shown in Tables 1, 2 and 3 for the distribution volume of the total component fraction (identical between methods), the fast component fraction, and the slow component distribution volume. Overall, the manual boundaries yielded better ICCs than the fixed boundaries, particularly for $\hbox {V}_{slow}$. The AI-generated boundaries had the same values as the manual boundaries as in this selection of participants and ROIs no errors occurred. For manual and AI-generated boundaries, good values of ICC were observed in ACGs, fusiform gyrus, and insula. The ICC was slightly lower in hippocampus, followed by the inferior frontal gyrus, with the occipital lobes showing very poor retest stability.

**Table 1:** ICC for $\hbox {V}_{slow}$, $\hbox {V}_{fast}$, $\hbox {V}_{total}$ of fixed boundaries

	$\hbox {V}_{slow}$	$\hbox {V}_{fast}$	$\hbox {V}_{total}$
ACGs	0.548	0.734	0.507
Fusiform gyrus	0.477	0.467	0.496
Hippocampus	0.226	0.191	0.679
Inferior frontal gyrus	0.178	0.652	0.305
Insula	0.603	0.625	0.527
Occipital lobes	0.000	0.000	0.000
Median	0.351	0.546	0.501

**Table 2:** ICC for $\hbox {V}_{slow}$, $\hbox {V}_{fast}$, $\hbox {V}_{total}$ of manual boundaries

	$\hbox {V}_{slow}$	$\hbox {V}_{fast}$	$\hbox {V}_{total}$
ACGs	0.804	0.683	0.507
Fusiform gyrus	0.895	0.915	0.496
Hippocampus	0.735	0.655	0.679
Inferior frontal gyrus	0.531	0.420	0.305
Insula	0.828	0.812	0.527
Occipital lobes	0.222	0.069	0.000
Median	0.770	0.669	0.501

**Table 3:** ICC for $\hbox {V}_{slow}$, $\hbox {V}_{fast}$, $\hbox {V}_{total}$ of AI-generated boundaries

	$\hbox {V}_{slow}$	$\hbox {V}_{fast}$	$\hbox {V}_{total}$
ACGs	0.804	0.683	0.507
Fusiform gyrus	0.895	0.915	0.496
Hippocampus	0.735	0.655	0.679
Inferior frontal gyrus	0.531	0.420	0.305
Insula	0.828	0.812	0.527
Occipital lobes	0.222	0.069	0.000
Median	0.770	0.669	0.501

## Discussion

      We have investigated whether boundary selection in band-pass spectral analysis can be automated with machine learning techniques. Overall, the Fine Decision Tree performed the best in this classification task, which we hypothesize is due to the relatively simple structure of our data, comprising only 101 channels, with most of the channel values being zero. Therefore, the dataset essentially represents high-dimensional sparse data.

KNN is affected by the distortion of distances caused by the majority of features being zero in sparse data classification. Moreover, KNN is not well-suited for high-dimensional data, leading to high computational complexity.

Fine Tree is inherently suitable for handling sparse data as it can ignore the majority of zero-valued features and focus only on features containing significant information. This effectively reduces the dimensionality of the dataset by ignoring the zero-valued features and efficiently utilizing the informative ones.

The accuracy of neural networks from MATLAB was relatively lower, at only around 85%, which may be due to overfitting. Neural networks can learn complex nonlinear relationships, but unlike the Fine Tree, they are not suitable for handling sparse data as they tend to overfit non-significant features, leading to performance degradation [42, 43]. Moreover, neural networks require a large amount of training data and computational resources, necessitating regularization and feature selection. The Fine Tree model has achieved a commendable level of accuracy in boundary selection. Additionally, it operates at a significantly faster speed compared to neural networks.

The accuracy of the 1D CNN fell between the Fine Tree and neural networks. This might be because CNNs excel at extracting local features; however, the non-zero feature channels in this dataset do not exhibit significant local patterns, possibly preventing CNN from fully leveraging its advantages [44, 45]. Additionally, the focus of this experiment was not on the architecture design and tuning of CNN, otherwise, CNN might have performed better.

In conclusion, Fine Tree was the most effective model for automated spectral boundary selection in our experiments as it efficiently handles and utilizes sparse features. Neural networks and CNN models could potentially perform better with specific tuning, such as further feature extraction, selection of important features, and the application of regularization techniques to better handle sparse data. However, the Fine Tree’s 96.92% accuracy is already quite satisfactory. Moreover, the fine tree model can significantly save time and computational resources, with its training time being an order of magnitude faster than that of a CNN.

There is still potential for improvement in our machine learning approach. In this dataset, the label with the most samples is the $1\textrm{st}$ bin out of 19 bins, while other labels have significantly fewer samples. There are no other labels after $19{\textrm{th}}$ bin because all subsequent bins are considered fast components. The imbalance with most samples in bin 1 increases the likelihood of the model failing to correctly classify minority bins and also raises the risk of overfitting.

We observed that the relative error was notably high. Based on the confusion matrix, this can be attributed to the fact that although the machine learning model demonstrates high accuracy, when errors do occur, the predicted results deviate significantly from the correct values, leading to large relative errors.

Based on the confusion matrix, there are 19 categories in total, therefore, there are a total of 19 possible locations representing slow components. This is because the slow component, representing the binding to $\alpha$5, was confined to the $1\textrm{st}$ bin through the $19{\textrm{th}}$ bin, while the faster bins were considered to represent the fast component. Most misclassifications occur at the $1\textrm{st}$ and $2{\textrm{nd}}$ bins, and the $18{\textrm{th}}$ and $19{\textrm{th}}$ bins, which represent the extreme locations of potential spectral boundaries. For ease of description, we define the $1\textrm{st}$ bin as the leftmost boundary and the $19{\textrm{th}}$ bin as the right boundary. Specifically, for boundaries that should be at the leftmost position, the model might classify them as being at the rightmost position, and vice versa (Fig. 5). We hypothesize that the reasons for misclassifications are:

1. Data Class Imbalance: There is a lack of sufficient training data for the rightmost boundary, while there is more data at the $1\textrm{st}$ bin compared to other bins. This imbalance increases the likelihood of the model selecting the $1\textrm{st}$ bin as the boundary, leading to errors.
2. Issues with Spectral Results: Spectral analysis results typically include both fast and slow components. Relatively slow fast components and relatively fast slow components may both appear around the $19{\textrm{th}}$ bin. This overlap can sometimes lead to misclassifications by the model. We calculated the probability of $\alpha$1 appearing within the $1\textrm{st}$ bin to $19{\textrm{th}}$ bin range, which was 12%.

To further improve the accuracy of boundary selection, a manual check of the spectral analysis results assigned to these boundaries could be performed. This is particularly important as, according to the confusion matrix, 43% of prediction errors occur at the $18{\textrm{th}}$and $19{\textrm{th}}$ bins.

The ICCs resulting from AI-generated boundaries are identical to those from manually annotated boundaries: Although the AI model has a 3.2% probability of error, these errors are subject-dependent, with 52% of classification errors occurring in only seven scans and predominantly in smaller ROIs; none of those occurred in the ICC dataset. In contrast, the ICC of fixed boundaries is significantly lower, which aligns with the findings described by Stokes et al. [26].

The Pearson correlation coefficients for within-subject between-regions and within-region between-subjects were 0.015 and 0.158, respectively, indicating that the model’s boundary selection has no significant correlation with either subjects or regions.

There are two possible reasons for low ICC of occipital lobes. First, the occipital lobes are a region with low $\alpha$5 subtype concentration, resulting in fewer slow peaks representing $\alpha$5. Consequently, the model may misidentify fast peaks as slow peaks, leading to incorrect spectral boundaries and subsequently affecting the generation of accurate parametric maps, which in turn impacts the ICC. Second, the test-retest sample size in this experiment is small, with only five pairs, and ICC calculations based on fewer than eight pairs should be interpreted with caution [46].

This study has some limitations. Firstly, the scanner used is not the most advanced, which means that the image resolution is limited. In the future, we plan to use more advanced scanners, which could potentially improve the accuracy of both spectral analysis [10] and parametric map generation. Secondly, the number of subjects in this study is relatively small. Small sample size can lead to a decrease in model accuracy and increase the risk of overfitting. In fact, due to the need for the metabolite-corrected parent plasma arterial input function, it is challenging to obtain a sufficiently large dataset. To address this limitation, we are also exploring ways to eliminate the reliance on arterial input functions, with image-derived input functions being a promising solution for the future.

## Conclusion

      The machine learning model developed provided accurate boundary predictions in 96.92% of regions, with minimal average bias. However, when errors occur, they can be large, owing to the sparsity of peaks. The automated method enables setting boundaries automatically for the vast majority of regions, offering the potential to accelerate analysis.

Band-pass spectral analysis can potentially be extended to other tracers that bind to more than two receptor subtypes and quantify the distribution of several receptor subtypes, e.g. of $\hbox {GABA}_A$ $\alpha$1/2/3/5 subtype binding using [$^{11}$C]flumazenil. Band-pass spectral analysis could also be extended to other systems where radiopharmaceuticals bind to more than one target, such as first-generation tau tracers such as [$^{18}$F]AV1451=T807=flortaucipir [47] or [$^{11}$C]yohimbine [48]

Additionally, the novel method developed in this study for generating regional parametric maps can produce maps by using boundaries predicted by the machine learning model across regions and subjects which is potentially more accurate than using a single boundary across subjects and regions. The approach allows for the intuitive visualization of the regional distribution of different receptor subtypes, enhancing the interpretation of data from PET tracers that bind to two or more receptor subtypes.

## Data Availability

Anonymized data are available from the corresponding author upon request.

## Abbreviations

PET: Positron emission tomography
GABA: Gamma-aminobutyric acid
CNN: Convolutional neural network
TAC: Time-activity curve
ROI: Region of interest
IRF: Impulse response function
CNS: Central nervous system
TLE: Temporal lobe epilepsy
MAPER: Multi-atlas propagation with enhanced registration
MICKPM: Modelling, input functions, compartmental kinetics and parametric maps software
$\hbox {V}_d$: Volume-of-distribution
$\hbox {V}_{total}$: Total component volume-of-distribution
$\hbox {V}_{fast}$: Fast component volume-of-distribution
$\hbox {V}_{slow}$: Slow component volume-of-distribution
ICC: Intraclass correlation coefficient
ACG: Anterior cingulate gyrus
SVM: Support vector machines
KNN: K-nearest neighbor
